# Variations in Postpartum Hemorrhage Management among Midwives: A National Vignette-Based Study

**DOI:** 10.1371/journal.pone.0152863

**Published:** 2016-04-04

**Authors:** A. Rousseau, P. Rozenberg, E. Perrodeau, C. Deneux-Tharaux, P. Ravaud

**Affiliations:** 1 Department of Obstetrics and Gynecology, Poissy-Saint Germain Hospital, Poissy, France; 2 INSERM U1153, METHODS (Méthodes en évaluation thérapeutique des maladies chroniques) Research Unit, Paris Descartes-Sorbonne Paris Cité University, Paris, France; 3 Research unit EA 7285, Versailles-St Quentin University, Saint Quentin en Yvelines, France; 4 Assistance Publique-Hôpitaux de Paris, Centre d’Epidémiologie Clinique, Hôpital Hôtel-Dieu, Paris, France; 5 INSERM U1153, EPOPé (Epidémiologie Obstétricale, Périnatale et Pédiatrique) Research Unit, Paris Descartes-Sorbonne Paris Cité University, Paris, France; Indiana University School of Medicine, UNITED STATES

## Abstract

**Objective:**

To assess variations in adherence to guidelines for management of postpartum hemorrhage (PPH) among midwives.

**Methods:**

A multicentre vignette-based study was e-mailed to a random sample of midwives from 145 maternity units in France. They were asked to describe how they would manage the PPH described in 2 case-vignettes. These previously validated case-vignettes described 2 different scenarios for severe PPH. Vignette 1 described a typical immediate, severe PPH and vignette 2 a less typical case of severe but gradual PPH. They were constructed in 3 successive steps and included multiple-choice questions proposing several types of clinical practice options at each step. An expert consensus defined 14 criteria for assessing adherence to guidelines issued by the French College of Obstetricians and Gynecologists in 2004 in the midwives’ responses. We analyzed the number of errors among the 14 criteria to quantify the level of adherence.

**Results:**

We obtained 450 complete responses from midwives from 87 maternity units. The rate of complete adherence (no error for any of the 14 criteria) was low: 25.1% in vignette 1 and 4.2% in vignette 2. The error rate was higher for pharmacological management, especially oxytocin use, than for non-pharmacological management and communication-monitoring-investigation. Adherence to guidelines varied substantially between and within maternity units, as well as between the vignettes for the same midwives.

**Conclusion:**

Reponses to case-vignettes demonstrated substantial variations in PPH management and especially individual variations in adherence to guidelines. Midwives should participate in continuous and individualized training.

## Introduction

Severe postpartum hemorrhage (PPH) is a leading cause of maternal mortality and morbidity worldwide [1–3] and occurs in around 1% to 2% of deliveries in high-income countries [3,4]. The incidence of PPH is increasing worldwide [5–8]: in the United States, the rate has increased from 2.3% to 2.9% (i.e., +26%) over the past 10 years [2,9]. Hemorrhage accounts for 12% of pregnancy-related deaths in the United States and 18% in France [10–13]. Moreover reports from confidential enquiries show that 67% of the US and 85% of the French deaths were avoidable [13–15], as they resulted from delay in treatment or inadequate management.

Furthermore, variations in clinical practice related to PPH occur between and within countries, even though national clinical practice guidelines for PPH are similar in France, other western European countries, the United States, and Canada [16–23]. Winter et al [16], using questionnaires to document policies of maternity units for the immediate management of postpartum hemorrhage in 12 countries, found considerable differences in the choice of pharmacological agents. Two French studies have showed that management of severe PPH is not optimal and that the guidelines are not fully applied [24,25]. The existence of variations in the conformity of clinical practice to guidelines between professionals in the same maternity units and between different PPH situations for the same professional has not yet been investigated, however, although it would be useful for developing effective strategies to improve clinical practices.

Clinical vignettes have been widely used to compare quality of clinical care and to assess practice variations across countries, health care systems, specialties, and clinicians [26–29]. The case-vignette method can be used to identify variations in practice and to understand discrepancies between guidelines and practices in PPH management. In a previous study, dynamic vignettes with several steps proved to be a valid tool that can accurately reflect real practices in such complex emergency situations as severe PPH [30]. The objective of our study was to assess variations in adherence to the guidelines issued by the French College of Obstetricians and Gynecologists [20,21] for management of postpartum hemorrhage (PPH) among midwives, who diagnose and provide initial management of PPH at the same time as they call for the obstetrician in some countries, including France and the United Kingdom. In France midwives have a specific medical education certified by a state diploma considered equivalent to a Masters degree, and midwifery is included in the Public Health Code as a medical professions along with doctors and dentists. Midwives work closely with the obstetricians and anesthesiologists on duty to manage life-threatening situations, notably severe PPH. They may prescribe some medications as well as oxytocin. Understanding the discrepancies between the guidelines and midwives’ actions during PPH should highlight the areas where improvement is needed.

## Material and Methods

This multicentre cross-sectional study was conducted from January to April 2014.

Midwives were requested to respond to an online survey, in which they answered multiple-choice questions about how they would manage 2 case-vignettes of PPH.

### Survey instrument: case-vignettes

Our previous validation study concerned 66 dynamic case vignettes that we developed to describe real incidents of severe PPH in several steps [30]. Briefly, vignettes were developed by abstracting from patient files women’s medical history and information about the pregnancy, labor, delivery, and PPH. All information that might identify the specific situation was intentionally changed. Cases were selected from Ile-de-France maternity unit birth registers according to the requirements of the validation study and included two cases each from each of 33 senior obstetricians.

Six obstetrics professionals (3 midwives and 3 obstetricians) jointly selected 2 case-vignettes among these 66 for this study. They opted for 2 very different situations: vignette 1 describes a typical immediate and severe PPH and vignette 2 a less typical case of severe but gradual PPH with constant trickle of blood (see S1 and S2 Files).

These 2 selected vignettes of PPH included multiple-choice questions that proposed different options for clinical care. We designed the vignettes to include 3 successive steps that re-created the course of each PPH. The first step included a partogram describing the medical history, labor, delivery and PPH. The next 2 steps of the vignette presented the postpartum course over the next 15 minutes (response to treatment) visually: bleeding was illustrated by photographs of simulated soaked pads and containers [31], and maternal condition by photographs of a simulated monitor display (pulse, blood pressure, and SpO_2_). At each step, midwives were asked how they would manage the emergency situation.

For each step, we used the same closed-ended questions–“What measures would you perform within the next 15 minutes”- for each of the three different types of management, for the midwife to choose none, one or more actions from the list of choices for each type of management (see S1 Text):

- pharmacological management: antibiotic, oxytocin, misoprostol (prostaglandin E1 analogue), methylergometrine, sulprostone (prostaglandin E2 analogue), tranexamic acid;
- non-pharmacological management: abdominal ultrasound, uterine massage, bimanual uterine compression, torsion of the cervix, bladder catheterization, manual examination of the uterine cavity, cervical examination with speculum, perineal repair, intrauterine tamponade, selective arterial embolization, surgical treatment;
- communication, monitoring and investigation: alert other members of the team, venipuncture for blood sampling, resuscitation and monitoring

When the midwife selected some interventions, the result in terms of patient response was mentioned in the next step in order to guide the next management decision. After responding to the questions at each step, participants could not return to the previous step to change their answers.

### Survey administration

In France, all maternity units, both public and private, belong to a regional perinatal network that groups together level 1 (no facilities for nonroutine neonatal care) and level 2 (with a neonatal care unit) units around one or more level 3 units (reference centres with an onsite neonatal intensive care unit). We selected 15 perinatal networks, about half the total number of networks in France. All 215 maternity units of 15 perinatal networks were eligible. Two networks (i.e., 37 maternity units) decided not to participate. Among the 13 participating networks, 33 units decided not to participate or were closed before our study started. Accordingly, our sample included 145 maternity units representing 27% of French maternity units.

We sent an email to the supervising midwife in each unit, explaining the aim of the survey and asking each to transmit the link to the survey website by email to all midwives who did worked during a arbitrarily selected period (from January 13 to 19 (Monday to Sunday), 2014) in the unit’s delivery room. If the midwives did not respond to the survey, their supervisors received two gentle email reminders 2 weeks apart [32].

### Main outcome

Criteria for assessing responses were determined in a two-step procedure involving two separate expert committees. The first comprised 3 midwives and 3 obstetricians previously involved in developing French guidelines for PPH or conducting studies on this topic. They were asked to respond to the 2 vignettes according to guidelines published by the French College of Obstetricians and Gynecologists in 2004 and updated in 2014[20–21], which are similar to those of both the American College of Obstetricians and Gynecologists [19] and the Royal College of Obstetricians and Gynaecologists [22]. A second committee of one obstetrician, one midwife, and one epidemiologist reviewed their answers and selected as criteria only those responses selected by all members of the first committee. Finally, 14 criteria were used to define adherence to guidelines for each vignette: 3 for pharmacological management, 8 for non-pharmacological management, and 3 criteria for other management (communication, monitoring and investigation) (Table 1): some were answers that had to be chosen, while others were answers that were always wrong in that circumstance. The remaining responses were considered neither correct nor incorrect and did not count in the assessment.

**Table 1:** Criteria for evaluation of adherence to guidelines.

**Pharmacological management:**
First line uterotonic: oxytocin in step 1
Second line uterotonic: sulprostone (prostaglandin E2 analogue) in step 2
No misoprostol (prostaglandin E1 analogue) in each step
**Non-pharmacological management:**
Manual placental delivery, manual examination of the uterine cavity in step 1
No intrauterine tamponade in step 1
No torsion of the cervix in step 1
Uterine massage in steps 1 or 2
Cervical examination with speculum in steps 1 or 2
No surgical treatment in steps 1 or 2
No selective arterial embolization in steps 1 or 2
Surgical treatment, selective arterial embolization and/or intrauterine tamponade in step 3
**Communication, monitoring and investigation:**
Alert other members of the team in steps 1 or 2
Venipuncture with blood count, hemostasis in steps 1 or 2
Resuscitation measure in steps 1 or 2

Management was considered appropriate when all 14 criteria were met. Finally, to quantify adherence to guidelines, we assessed the number of errors, defined as the number of the 14 selected criteria performed incorrectly, that is, the actions that should not have been taken and the failure to take necessary actions. Thus 0 to 14 errors were possible for each vignette. Adherence was assessed separately for each vignette (expected responses were, however, identical).

### Ethics statement

Our institutional review board (Comité de Protection des Personnes Ile de France Paris- XI) approved this study on September 13, 2012, as number 12066.

Participants were all midwives who completed a questionnaire about how they would respond to 2 clinical vignettes. By clicking on the survey link and completing the questionnaire, they provided informed consent to participate. Participants were informed about the purpose of the study at the beginning of the study (through the email that led them to contact the study site and by the introduction to the study).

### Statistical analysis

Data are available in S1 Table.

Characteristics of midwives and maternity units and adherence to guidelines were described with means and standard deviation (SD) for quantitative variables and frequencies and percentages for qualitative variables.

To compare adherence to guidelines between vignette 1 and vignette 2, numbers of errors for the 3 types of management were compared with Wilcoxon signed rank tests.

To evaluate the correlation of adherence to guidelines between midwives in the same maternity units, we calculated intraclass correlation coefficients (ICC) for error counts for pharmacological management, non-pharmacological management and other management, separately for each type of management.

Finally, we assessed the correlation between error counts in vignette 1 and vignette 2 with Pearson’s correlation coefficient.

All statistical tests were two-sided, and *P* < .05 was considered statistically significant. Statistical analysis was conducted with R statistical software, version 3.0.1.

## Results

**Fig 1:**
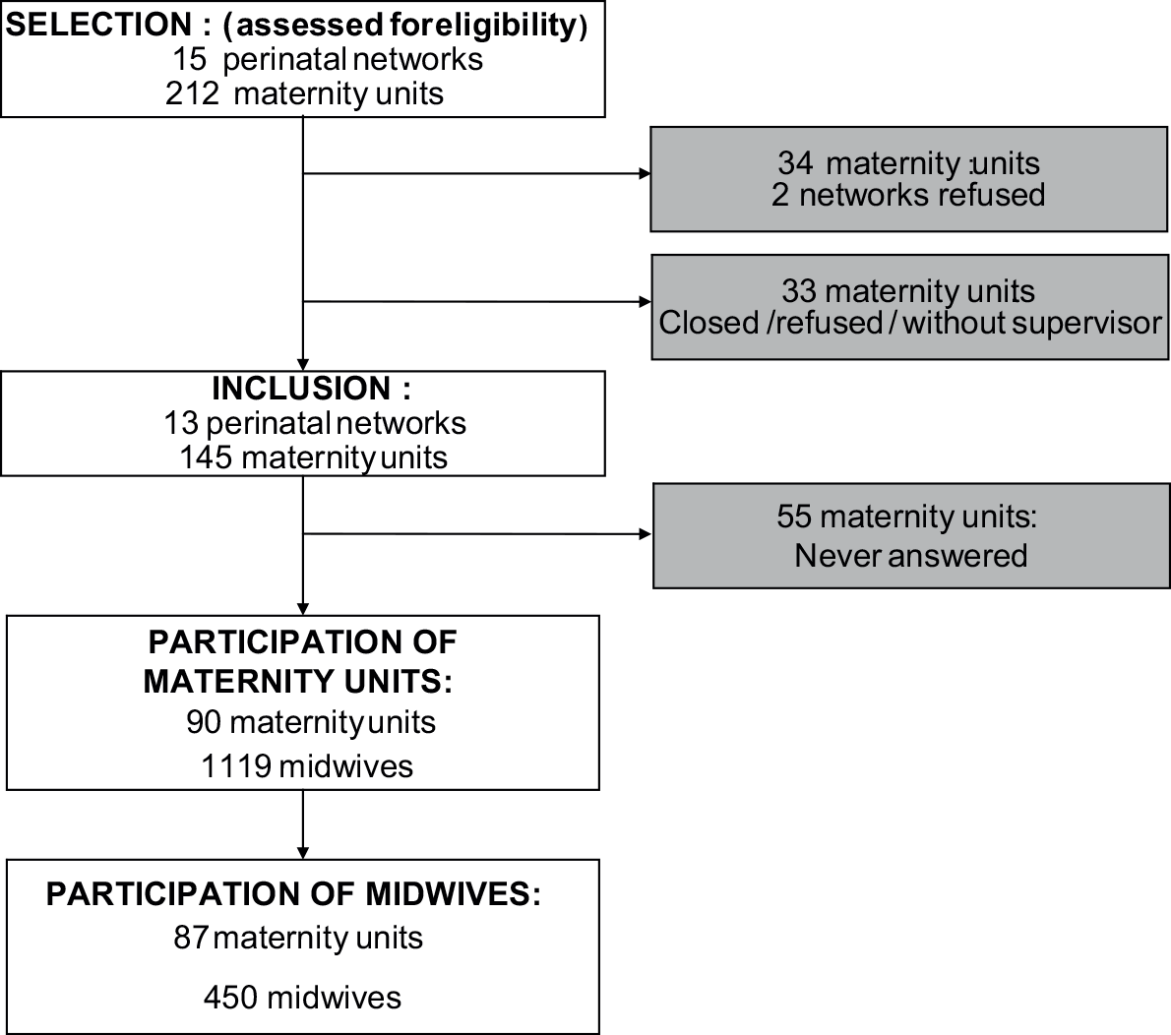
Flowchart. This figure corresponds to the flowchart of the study.

We obtained complete responses from 450 midwives from 87 maternity units (Fig 1).

The mean (+/-SD) age of midwives was 34.72 years (+/-8.44) and 94.4% were women. Table 2 summarizes the characteristics of the maternity units.

**Table 2:** Characteristics of maternity-units and midwives.

**Maternity units**		**n = 87**
Public, n (%)		65 (74.7)
University, n (%)		14 (16.1)
Level of care, n (%)	level 1	35 (40.2)
	level 2	36 (41.4)
	level 3	16 (18.4)
Volume of births per year	mean (SD)	1623.2 (997.46)
	median [25th, 75th centile]	1362.5 [803 ; 2257.83]
**Midwives**		**n = 450**
Gender: Female, n (%)		425 (94.4)
Age, year, mean (SD)		34.72 (8.44)
Experience*, year, mean (SD)		11.38 (8.71)

* Experience corresponds to number of years of professional experience after completion of midwifery school.

All midwives reported that their institution has a specific protocol for PPH management, and 81.1% that they know the guidelines issued by the French College of Obstetricians and Gynecologists.

### Description of adherence to guidelines

For vignette 1, 113 (25.1%) midwives chose appropriate management that complied completely with guidelines (no errors for any of the 14 criteria), 230 (51.1%) at least 13 correct answers (only 1 error), and 315 (70.0%) at least 12. For vignette 2, 19 (4.2%) midwives respected all 14 criteria (0 errors), 84 (18.6%) proposed at least 13 correct answers, and 170 (37.7%) at least 12 (Table 3). Among midwives who had only one error, the most common one was the failure to administer oxytocin: 41.9% in vignette 1 and 30.8% in vignette 2.

**Table 3:** Adherence to guidelines and number of correct answers.

	Vignette 1, n (%)	Vignette 2, n (%)
14 criteria met (0 error)	113 (25.1)	19 (4.2)
13 criteria met (1 errors)	117 (26.0)	65 (14.4)
12 criteria met (2 errors)	85 (18.9)	86 (19.1)
11 criteria met (3 errors)	70 (15.6)	70 (15.6)
10 criteria met (4 errors)	45 (10.0)	73 (16.2)
9 or fewer criteria met (5 errors and more)	20 (4.4)	137 (30.5)

**Fig 2:**
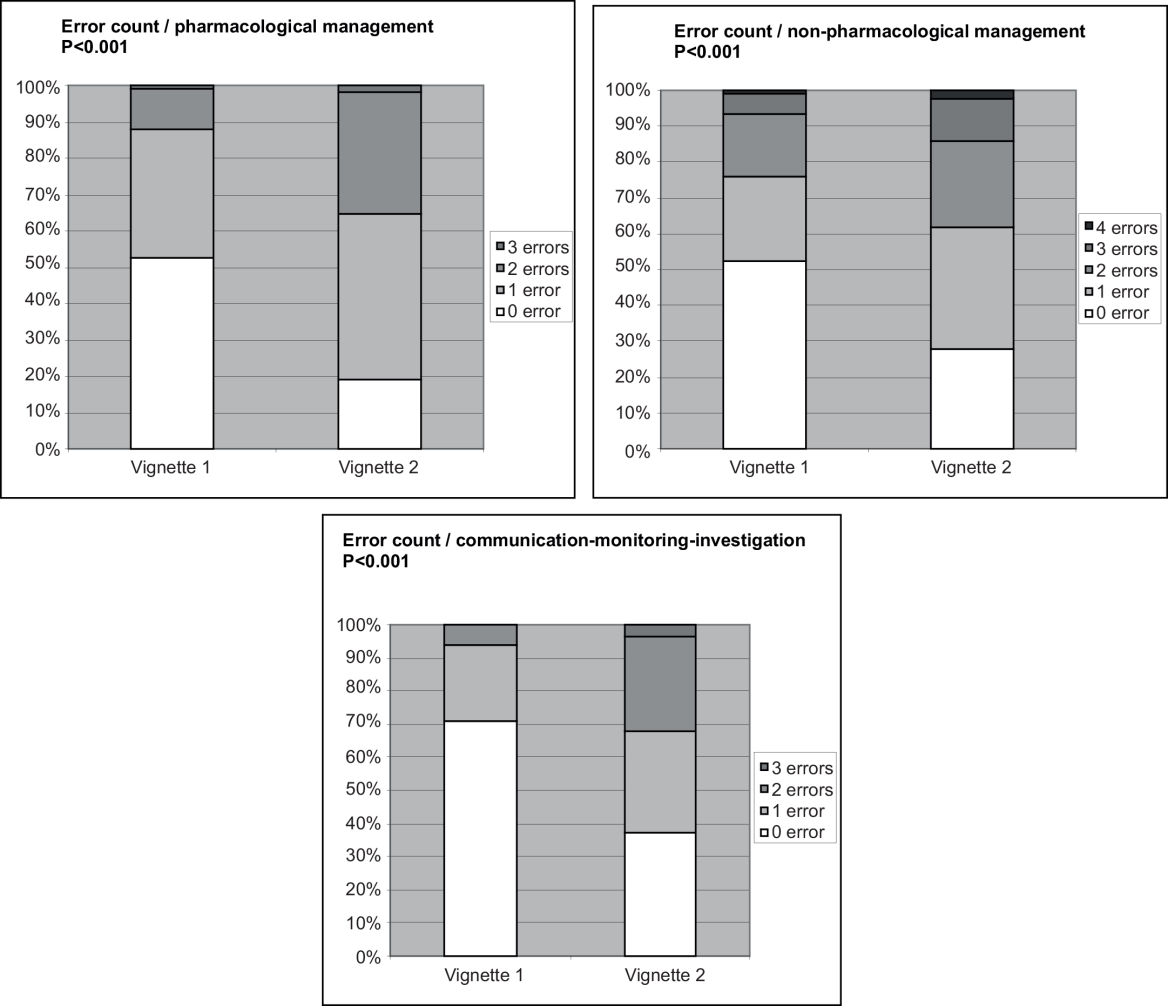
Difference in adherence to guidelines between vignette 1 and vignette 2 for the 3 types of management. This figure indicates the adherence to guidelines for vignette 1 and vignette 2 regarding pharmacological management, non-pharmacological management and communication-monitoring-investigation.

For vignette 1, pharmacological management was appropriate and completely consistent with guidelines in 237 responses (53.7%), non-pharmacological management in 235 (52.25%), and communication-monitoring-investigation in 319 (70.9%); for vignette 2, however, these figures were significantly poorer for each type of management (*P*<0.001 for each) substantially lower: 86 (19.1%), 126 (28%), and 168 (37.3%), respectively (Fig 2).

As the results above indicate, adherence to guidelines was better for vignette 1 than for vignette 2. Adherence to each criterion in vignette responses 1 and 2 is detailed in Table 4. The use of oxytocin was the correct action least frequently selected.

**Table 4:** Adherence to 14 criteria in vignette response.

	Vignette 1, n (%)	Vignette 2, n (%)
**Pharmacological management:**		
First line uterotonic: oxytocin in step 1	291 (64.7)	201 (44.7)
Second line uterotonic: sulprostone (prostaglandin E2 analogue) in step 2	348 (77.3)	181 (40.2)
No misoprostol (prostaglandin E1 analogue) in each step	440 (97.8)	436 (96.9)
**Non-pharmacological management:**		
Manual placental delivery, manual examination of the uterine cavity in step 1	444 (98.7)	326 (72.4)
No torsion of the cervix in step 1	450 (100)	448 (99.6)
No intrauterine tamponade in step 1	440 (97.8)	445 (98.9)
Uterine massage in steps 1 or 2	436 (96.9)	296 (65.8)
Cervical examination with speculum in steps 1 or 2	403 (89.6)	402 (89.3)
No surgical treatment in steps 1 or 2	418 (92.9)	443 (98.4)
No selective arterial embolization in steps 1 or 2	366 (81.3)	410 (91.1)
Surgical treatment, selective arterial embolization and/or intrauterine tamponade in step 3	286 (63.6)	260 (57.8)
**Communication, monitoring and investigation:**		
Alert other members of the team in steps 1 or 2	445 (98.9)	411 (91.3)
Venipuncture with blood count, hemostasis in steps 1 or 2	353 (78.4)	249 (55.3)
Resuscitation measure in steps 1 or 2	393 (87.3)	247 (54.9)

### Variations in adherence to guidelines

**Fig 3:**
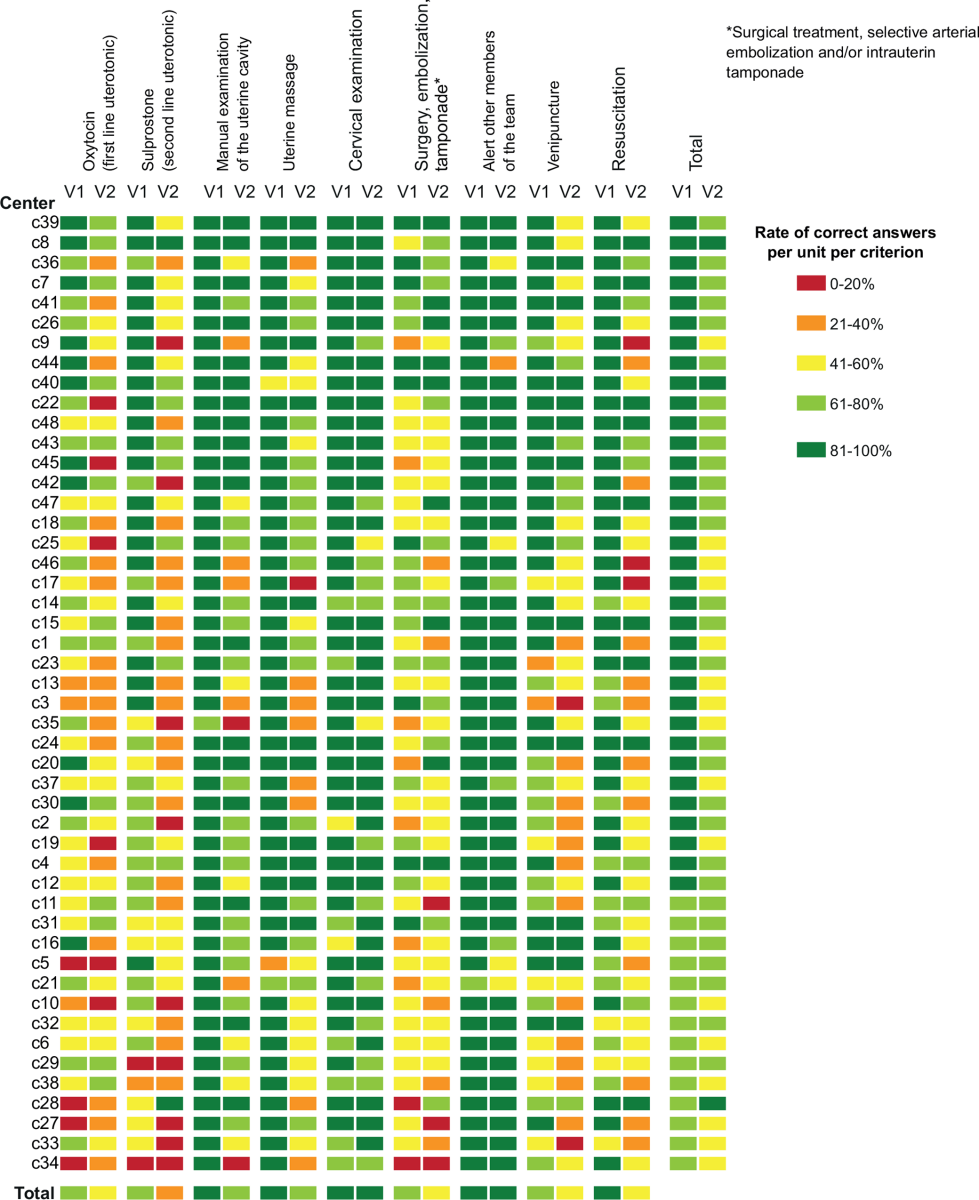
Adherence to guidelines between and within maternity units, between the 2 vignettes. This figure indicates the rates of correct answers for the 9 most important criteria and for maternity units with more than 4 participating midwives. Each line represents a maternity unit, and they ranked in descending order of global adherence.

Fig 3 reports the variations in adherence to guidelines between maternity units, between midwives within each maternity unit, and between the 2 case-vignettes. Again, adherence was lower for vignette 2 than for vignette 1 and was lowest for oxytocin use.

We observed a centre effect, especially for vignette 1: the ICC for error counts for pharmacological management was 0.19 [95% CI: 0.10; 0.29], which indicates that 19% of the overall variance in error counts may be explained by factors at the level of the maternity units (centre effect) and 81% to midwife-level or unknown factors. The ICC for error counts for non-pharmacological management was 0.08 [0.01; 0.17], and for communication-monitoring-investigation 0.07 [0; 0.16]. For vignette 2, the ICC for error counts for pharmacological management was 0.07 [0; 0.16], for non-pharmacological management 0.06 [0; 0.14], and for communication-monitoring-investigation 0.10 [0.03; 0.20].

The correlation between the error counts in vignette 1 and vignette 2 was low but still significant: Rho: 0.31 [0.22–0.39] and indicates that midwives tended to make fewer errors for vignette 2 when they made fewer errors for vignette 1.

## Discussion

### Principal findings of the study

The global rate of complete adherence to guidelines was low: 25.1% in vignette 1 and 4.2% in vignette 2. It was lower for pharmacological management, especially oxytocin use, than for either non-pharmacological management or communication-monitoring-investigation. Midwives recognized the severity of the PPH by calling the team but many did not provide pharmacological management sufficiently promptly. Our study showed variations in adherence to guidelines between and within maternity units and between the two different PPH situations.

### Clinical meaning of the study

We noted that management was poorer for vignette 2 than for vignette 1. There are two main explanations for this difference. First, the PPH situation in vignette 2 is probably less common; secondly, and perhaps relatedly, the guidelines are probably less appropriate or less useful for gradual, slow hemorrhages with a constant trickle of blood, so that midwives are uncertain about its optimal management.

Even though most midwives (81.1%) reported that they knew the French guidelines, our study showed poor adherence with them. It has already been demonstrated that physicians frequently fail to follow clinical practice guidelines [33]. Lack of awareness, familiarity or agreement have been suggested as possible barriers to guideline adherence. Professionals appear to know that there are guidelines but either do not know the contents or prefer more conservative measures than those recommended by guidelines [33]. The French guidelines are published by the French College of Obstetricians and Gynecologists and intended to be disseminated to every maternity unit and integrated into their protocol. We do not know either if all units actually include these guidelines in their protocols or if they in fact follow either the guidelines or the protocols. Moreover we do not know if pharmacological management is specified in the protocols. If midwives made errors in adherence to guidelines, we could not know if 1) they applied their protocol and it was not consistent with the national guidelines, or 2) they did not apply their protocol, which was consistent with national guidelines.

When we assessed each criterion separately (Table 4, Fig 3), the highest error rate concerned oxytocin. The other criteria with high error rates were surgery, selective arterial embolization and/or intrauterine tamponade, blood tests, and resuscitation measures, possibly because these decisions are beyond midwives’ expertise.

Winter et al [16] found similar results in a study by postal questionnaires of policies for immediate PPH management sent to maternity units in 12 European countries: European countries varied in pharmacological management and oxytocin use, but little in uterine massage rates.

Driessen et al [25] also studied initial care in a cohort of 4550 women with PPH due to uterine atony in 106 French maternity units. They found oxytocin administration inappropriate (because delayed by more than 10 minutes or not done at all) in 24.5% of cases. We approached these time-dependent actions by defining the criteria according to the steps, with oxytocin use expected, for example, within 15 minutes in step 1.

The delay or failure to use oxytocin that we observed may explain the high rate of invasive treatments reported by Kayem et al [34]. They found a rate of invasive second-line therapies for PPH significantly higher (by a factor of 6 to 8) in France than in the United Kingdom or the Netherlands.

Inappropriate oxytocin use, that is, failure to use it in a timely manner or at all, may result in less effective management of early stages of PPH, before it has become severe, and thus in a relatively higher proportion of cases that are not controlled at those stages. This delay may explain the high rate of maternal deaths from hemorrhage in France.

### Strengths and limitations of the study

Our study has a number of strengths. The characteristics of participating units were similar to those of French maternity units overall [35,36].

The case-vignette is a simple tool but no less valid than more complicated methods for evaluating adequacy of care and adherence to guidelines, as shown by the similarity of our results to those of previous studies using other methods [16,20,21]. Case-vignettes have been widely used to analyze practices such as screening, diagnosis, care, assessment of prognosis, and ethics in decision making. This tool allowed us to describe important variations in adherence to guidelines at the individual level, both between midwives and between 2 different situations of PPH managed by the same midwife. Few if any previous studies have shown variations in PPH management at the individual level.

This study also has limitations. Case-vignette is a theoretical approach, based on plans and intentions and not actual practice. A vignette cannot instill the sense of urgency and stress generated by PPH. Nor can it adequately transcribe the multidisciplinary approach that is necessary to improve care and that probably decreases both omissions and errors. Theoretical approaches testing reflection rather than action are also likely to be subject to a social desirability bias that may result in overestimating appropriate management and adherence to guidelines. Evaluating clinical practice with a clinical vignette and a multiple-choice rather than an open-ended format also tends to overestimate participant performance [37]. Finally, the midwives who participated in our sample were probably those the most interested in the topic and in quality of care or continuing education and were therefore more likely to be able to respond correctly. Nonetheless, given the low level of complete adherence, we may wonder if this limitation played any role in our findings.

Appropriate management was defined by complete adherence to all criteria—selecting every required answer and not selecting any wrong answers. Expecting strict adherence to all 14 criteria is probably unrealistic; and management may well finally prove to be appropriate without complete and strict adherence to all 14 of the guideline-based criteria. Therefore we also assessed the number of errors separately for each vignette and each criterion separately. Finally each criterion had the same weight in our study. Undoubtedly some criteria are more essential to adherence to guidelines than others. Several studies have shown the efficacy of oxytocin [38], which is recommended in all guidelines [23], while the importance or value of uterine massage has never been demonstrated [39]. That is part of the reason that we assessed the number of errors overall and individually by criterion.

Finally, this study only involved midwives. The results cannot be generalised to obstetricians or general (or family) practitoners. Moreover, even for midwives, generalization may be possible only in countries where midwives provide initial management of PPH.

## Conclusion

Case vignettes were effective for demonstrating variations in adherence to guidelines for PPH management, especially at the individual level. Midwives appropriately alert other team members. However, their knowledge about the indications, route and dosage of oxytocin, which is the firstline uterotonic treatment requires improvement. The center effect found in our study shows the need to continue efforts in each center to improve department protocols, training, and morbidity and mortality review, especially among midwives. Clinical vignettes are a useful tool for measuring quality. They can be used in each institution to identify individual discrepancies with good practices and to identify the training necessary for improvement, e.g. simulation. Different factors may be considered to explain discrepancies with guidelines: those related to individual characteristics of parturients, those related to medical care and both personal and professional factors related to health care providers. These factors should be explored.

## Supporting Information

S1 File Vignette 1. (PDF)

S2 File Vignette 2. (PDF)

S1 Table Database of midwives’ responses. (XLS)

S1 Text Exhaustive list of available choices for multiple-choice questions. (PDF)
