# Supplementary material for: Variations in Postpartum Hemorrhage Management among Midwives: A National Vignette-Based Study
Source: PLoS One. 2016 Apr 4;11(4):e0152863. doi: 10.1371/journal.pone.0152863 (PMC4820253; doi:10.1371/journal.pone.0152863)
Supplement: S1 Text — (PDF) [file pone.0152863.s004.pdf]

Pharmacological management

□ antibiotic

□ Cytotec® : misoprostol
- □ per os □ intrarectale
- □ 50 µg □100 µg □200 µg
- □400 µg □600 µg □800 µg

□ Exacyl® : tranexamic acid intravenous
- □ 500mg □ 1g
- □ 1.5g □ 2g
- □ 2.5g

□ Methylergometrine intramuscular
- □ 0.1mg □ 0.2mg

□ Nalador® : sulprostone intravenous during 1h
- □ 100µg □ 200µg □ 300µg
- □ 400µg □ 500µg □ 600µg

□ Syntocinon® : oxytocin
- □ intravenous push (bolus) :
  - □ 5UI □ 10UI □ 15UI □ 20UI
- □ slow intravenous (1min) :
  - □ 5UI □ 10UI □ 15UI □ 20UI
- □ intravenous infusion :
  - □ 5UI □ 10UI □ 15UI □ 20UI
- □ flow acceleration of the current infusion

□ Other:

□ None

Non-pharmacological management

□ Surgical treatment considered

□ Bimanual uterine compression

□ Manual examination of the uterine cavity

□ Abdominal ultrasound

□ Selective arterial embolization considered

□ Uterine massage

□ Cervical examination with speculum

□ Perineal repair

□Bbladder catheterization

□ Intrauterine tamponade considered

□ Torsion of the cervix

□ Other:

□ None

Communication, monitoring and investigation

□ Alert other member of the team
- □ obstetric team
- □ anesthesiologist team

□ Venipuncture for blood sampling
- □ coagulation factors
- □ liver function tests
- □ haemostasis
- □ serum electrolytes
- □ blood counts including platelet counts

□ Resuscitation
- □ second intravenous access
- □ monitoring
- □ volume replacement
- □ other :

□ Other :

□ None
